# Analysis of Circular RNA-Related Competing Endogenous RNA Identifies the Immune-Related Risk Signature for Colorectal Cancer

**DOI:** 10.3389/fgene.2020.00505

**Published:** 2020-06-03

**Authors:** Wei Song, Jun Ren, Chuntao Wang, Yuhang Ge, Tao Fu

**Affiliations:** Department of Gastrointestinal Surgery II, Renmin Hospital of Wuhan University, Wuhan, China

**Keywords:** colorectal cancer, circRNA, competitive endogenous RNA, immune-related genes, prognostic signature

## Abstract

**Background:**

Recent papers have described circular RNAs (circRNAs) playing important roles in the development and progression of colorectal cancer (CRC). However, the expression profiles of circRNAs and their functions in CRC have rarely been studied. The objective was to identify circRNAs involved in the carcinogenesis and progression of CRC and to explore potential molecular mechanisms as a competitive endogenous RNA (ceRNA). Moreover, we aimed to establish an immune-related gene signature for predicting the overall survival (OS) of CRC.

**Methods:**

The expression patterns of circRNA, miRNA, mRNA, and clinicopathological data were collected from the GEO and TCGA databases. A ceRNA network would be established, and the functional enrichment analyses were performed. The protein-protein interaction network (PPI) was constructed, and hub genes were identified using a cytohub plugin. Subsequently, an immune-related signature was developed based on mRNAs in the ceRNA network. In addition, OS-nomogram was constructed by combining an immune-related signature and clinicopathological characterization to predict the OS.

**Results:**

We established a circRNA-miRNA-mRNA ceRNA network. Kyoto Encyclopedia of Genes and Genomes (KEGG) pathway analysis revealed that the mRNAs were mainly enriched in neuroactive ligand-receptor interaction, Wnt signaling pathway, cell adhesion molecules (CAMs), and renin secretion. PPI network and module analysis identified 10 hub genes, and the circRNA-miRNA hub gene regulatory modules was established. After univariate and multivariate analysis, seven immune-related genes in the ceRNA network were used to construct the immune-related signature. Patients were divided into low-risk and high-risk groups, and there were significant differences in the OS. The ROC of the nomogram indicated the satisfactory accuracy and predictive power. Furthermore, we established a prognostic nomogram based on immune-related risk score and clinical characterization. The ROC and calibration curves revealed the accuracy of the nomogram. In addition, the high-risk score was positively correlated with six immune infiltrating cells (*P* < 0.05).

**Conclusion:**

We screened the key genes and established a circRNA-related ceRNA network involved in CRC, which will assist in understanding the molecular mechanisms underlying the carcinogenesis and progression. Moreover, our proposed immune-based signature may predict survival and reflect the immune status of CRC patients.

## Introduction

Colorectal cancer (CRC) is one of the most commonly diagnosed malignancies and one of the leading causes of death from cancer worldwide (Bray et al., 2018). The incidence of CRC is expected to increase to 160% by 2030, with an estimated 2.1 million new cases and 1.1 million deaths per year (Arnold et al., 2017). Considerable progress has been made in in the comprehensive treatment modalities of CRC over the past few decades. However, due to its late detection and high recurrence rate, mortality remains high, and the OS remains poor (Roxburgh and McMillan, 2012; Ludmir et al., 2017). Thence, it is essential to explore more reliable markers to precisely stratify the risk of individual patients and to implement personalized treatment for CRC patients.

Circular RNAs (circRNAs) are a new identified class of noncoding RNAs with a covalent loop structure without the 5′-end cap and 3′−end ploy A tail. They can prevent their degradation by RNA exonuclease and confer a strong stability in specific cells (Wang et al., 2016). Numerous studies have indicated that abnormal expressions of circRNAs may be a potential mechanism for the development and progression of cancer (Braicu et al., 2019; Wu et al., 2019). Although the function mechanism of circRNAs remains unclear, accumulating evidence suggest circRNAs may completely bind with miRNAs to influence the interaction between miRNAs and mRNAs and contribute to the progression of tumors, which was called as competitive endogenous RNA (ceRNA) hypothesis (Salmena et al., 2011). For example, has_circ_0009361 was shown to be downregulated in CRC tissues. Mechanistically, has_circ_0009361 may sponge miR-582 to regulate adenomatous polyposis coli 2 (*APC2*) level to inhibite cell proliferation, EMT, migration, and invasion (Geng et al., 2019). Similarly, hsa_circ_0008035 can interact with *YBX1* by the sponging of miR-375 in gastric cancer (Huang et al., 2019). However, the tumor microenvironment is very complex, and it is not ideal to research one or several relationships of ceRNA in isolation.

Currently, postoperative adjuvant therapy and prognosis were based on the comprehensive evaluation of the TNM staging system (Amin et al., 2017). However, it provides only limited information for the clinical prognostication, since even patients within the same stage display a strong heterogeneity for prognosis and treatment response. In addition, this system cannot predict the prognosis of individual patients. Therefore, it is necessary to continuously develop better methods to more accurately predict the survival of CRC. Recently, some nomograms have been widely used for predicting the prognosis of CRC (Fernández Montes et al., 2019; Qu et al., 2019). For example, Fernández Montes et al. (2019) developed a nomogram to predict 3-, 6-, and 12-month OS based on six variables, including the tumor site, ECOG PS, *BRAF* mutations, the number of metastatic sites, the response to first-line, and CEA. Despite significant progress in these studies, few have considered the use of genetic characteristics to construct a risk stratification system of CRC.

In the present study, we established the circRNA ceRNA network related to CRC according to bioinformatic prediction. From the ceRNA network, we identified immune-related genes and established an immune-related risk signature. In addition, the signature was assessed in the receiver operating characteristic (ROC) analysis. Finally, a nomogram containing an immune-related gene and clinicopathological features had also been established to evaluate the probability of survival rate in CRC patients.

## Materials and Methods

### Data Acquisition

We downloaded the circRNA (GSE126095) gene expression profiles from the GEO database^1^. The dataset included 10 CRC tissues and 10 paired normal colorectal tissues. The data of miRNA-seq (539 CRC samples and nine normal samples), mRNA-seq (571 CRC samples and 44 sample tissues), and their clinical data of CRC patients was collected from the Cancer Genome Atlas^2^. Immunologically relevant list of genes was downloaded from the IMMPORT Database^3^. Given that all data were downloaded from the public databases, the current study was no required approval by ethics committee.

### Screening of DEGs

The “limma” and “edgeR” package was used to identify differentially expressed genes (DEGs). Based on the cut-off value, adjusted *P*-values < 0.01, and |log fold change (FC)| > 3 were used for selecting differentially expressed circRNAs (DEcircRNAs). The miRNAs and mRNAs that met the cut-off criteria of the adjusted *P*-values < 0.05 and |logFC| > 2 were screened out as differentially expressed miRNAs (DEmiRNAs) and mRNAs (DEmRNAs).

### CeRNA Network Construction

We used the Cancer-Specific CircRNA^4^ to screen the target miRNAs of the DEcircRNAs. These target miRNAs were further screened by the DEmiRNAs obtained from TCGA database. Then, the target genes of these DEmiRNAs were predicted using the TargetScan, miRDB, and miRTarBase databases (Fromm et al., 2015; Wong and Wang, 2015). Only the target genes predicted by these three databases were retained, which were then overlapped with the DEmRNAs collected from the TCGA to obtain the CRC associated miRNA-mRNA interaction pairs. Finally, according to the predicted relationship of circRNA-miRNA and miRNA-mRNA, the ceRNA network was constructed using the Cytoscape software^5^.

### Functional Enrichment Analysis

To screen the possible function of mRNAs in ceRNA network, we performed the Gene Ontology (GO) function and the Kyoto Encyclopedia of Genes and Genomes (KEGG) pathway enrichment analysis by “clusterProfiler” package in R. *P* < 0.05 was considered statistically significant.

### PPI Network Construction and Identification of Hub Genes

We used the Search Tool for the Retrieval of Interacting Genes (STRING)^6^ database (Wang et al., 2011) to establish the PPI network of DEmRNAs. In this study, we used interaction score greater than 0.9 as the cut-off criterion. Subsequently, the hub genes were identified by cytoHubba plug in the Cytoscape software (v 3.7.0).

### Signature Development and Validation

To identify differentially expressed immune-related genes, we downloaded immune-related genes list, and intersected with the identified DEmRNAs in the ceRNA network.

Only the shared genes were left. To discover potential immune-related genes that are significantly associated with the OS, we conducted the univariate Cox proportional hazards regression. Possible immune-related genes with a *P* < 0.05 from univariate analysis were further included in a multivariate Cox regression analysis backward stepwise regression. An immune-related signature was established by multivariate Cox regression analysis. The regression coefficients (β) of each related gene are reserved for the development of immune-related signature. The risk score = ∑*exp*^(*mRNA*)*^β. By the median risk score as the cut-off value, the patients were divided into high and low risk groups. The Kaplan-Meier survival analysis, time-dependent receiver operating characteristic (tdROC) curve analysis, and calibration curve analysis were used to evaluate predictive ability of the signature.

### Construction and Validation of Nomogram

Based on the immune-related risk score and clinicopathological parameters, a prognostic nomogram was established by the “rms” software package in R. Discrimination between the outcomes of observations and predictions using the area under the curve (AUC) of ROC in the package of “survivalROC” in R. The value of the AUC was range 0.5 from 1.0, with 0.5 indicating a random probability, and 1.0 indicating the perfect ability. A calibration map was generated by comparing the nomogram prediction probability and the observation for the 3-year and 5-year OS rates. In addition, we compared predictive accuracy between the nomogram and a single independent factor by the ROC curve. The R software (version: 3.5.3) and SPSS 23.0 (Chicago, IL, United States) were applied to conduct all the analyses. Data was considered to be statistically significant when *P* < 0.05.

### Estimation of Tumor-Infiltrating Immune Cell Types

Tumor Immune Estimation Resource TIMER,^7^ was used to analyze the correlation between tumor-infiltrating immune cells signatures and risk score. TIMER can systematically analyze immune infiltration of various types of cancer. The abundance of the six immune infiltrates was estimated by statistical methods and validated using pathological estimates. The six immune cells are composed of CD4+ T cells, CD8+ T cells, B cells, Macrophages, Neutrphils, and Dendritic cells.

## Results

### Identification of DEGs in CRC Patients

**FIGURE 1:**
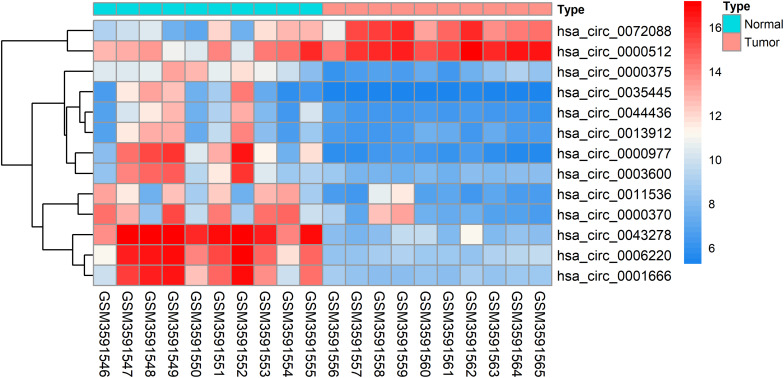
Heatmap analysis of differentially expressed circRNAs.

The DEcircRNAs were identified by the “limma” package in R (adjusted *P* < 0.01 and |logFC| > 3). In total, 13 DEcircRNAs that included two upregulated circRNAs and 11 downregulated circRNAs were screened (Figure 1). Using the criteria of adjusted *P* < 0.02 and |logFC| > 2, 381 DEmiRNAs (including 276 upregulated one and 105 downregulated ones), 3,933 DEmRNAs (including 2644 upregulated one and 1289 downregulated ones) were identified. A total of 2,498 immune-related genes were downloaded from the IMMPORT Database. We identified 91 differentially expressed immune-related genes of adjusted *p*-value < 0.05 and |logFC| > 2, of which, 42 genes were down-regulated, and 49 genes were up-regulated. The clinicopathologic characteristics of CRC patients are summarized in Table 1.

**TABLE 1:** Clinicopathologic characteristics of CRC patients.

**Variables**	**Case, *n* (%)**
Age (M ± SD, years)	65.81 ± 12.45
Gender	
Female	241 (46.0)
Male	283 (54.0)
History of polyps	
No	309 (59.0)
Yes	146 (27.9)
Unknown	69 (13.2)
Presentation o metastases	
No	26 (5.0)
Yes	455 (86.8)
Unknown	43 (8.2)
Microsatellite instability	
Yes	8 (1.5)
No	86 (16.4)
Unknown	430 (82.1)
Tumor location	
Colon	451 (86.1)
Rectum	73 (13.9)
Lymphatic invasion	
No	284 (54.2)
Yes	189 (36.1)
Unknown	51 (9.7)
Venous invasion	
No	345 (65.8)
Yes	110 (21.0)
Unknown	69 (13.2)
Stage	
I	98 (18.7)
II	199 (38.0)
III	143 (27.3)
IV	84 (16.0)
Response to first-line therapy	
Complete response	146 (27.9)
Other	41 (7.8)
Unknown	337 (64.3)

*Other: partial response, progressive disease, and stable disease.*

### Construction of ceRNA Network

**FIGURE 2:**
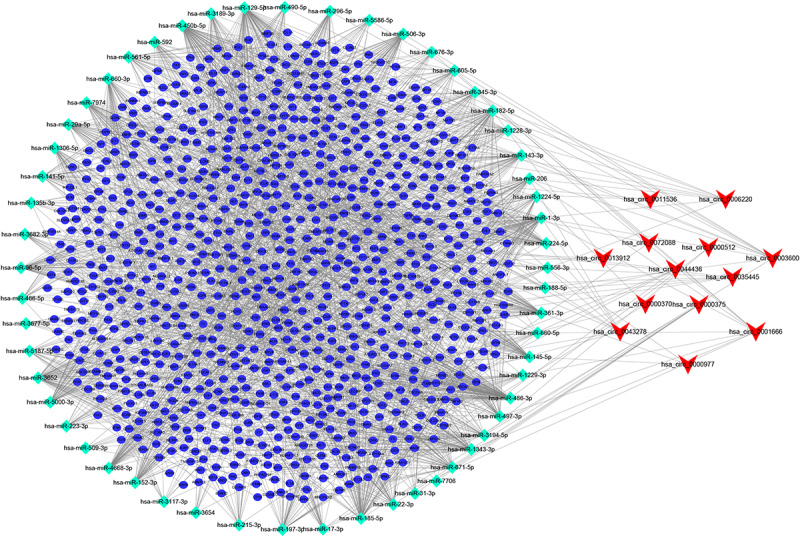
circRNA-miRNA-mRNA ceRNA network in CRC patients. V, diamonds, and ellipses represent circRNAs, miRNAs and mRNAs, respectively.

After searching the CSCD database, we identified 713 circRNA-miRNA pairs, including 13 DEcircRNAs and 549 miRNAs. After intersecting with the 381 DEmiRNAs, only 57 miRNAs remained. Subsequently, the target genes were predicted for these 57 miRNAs by TargetScan, miRDB, and miRTarBase databases, which were then overlapped with 3,933 DEmRNAs collected from the TCGA, resulting in 864 genes shared. On the basis of the combination of the above data, we established the ceRNA network (Figure 2).

### Functional Enrichment Analysis

**FIGURE 3:**
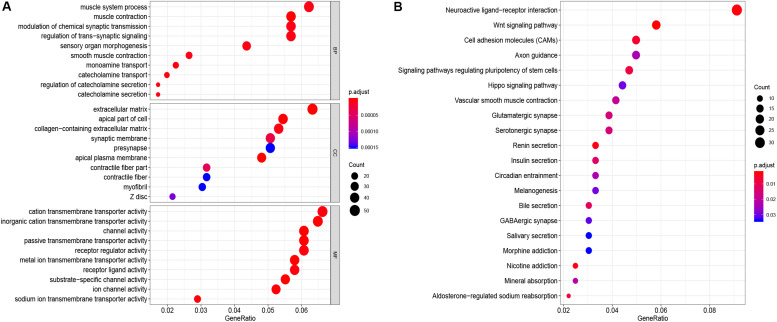
Enrichment of top 10 GO terms **(A)** and KEGG pathways **(B)** of differentially expressed mRNAs. The node color changes gradually from blue to red in ascending order according to the adjust *p*-values. The size of the node represents the number of counts.

GO function annotation of the 864 DEmRNAs includes three parts: biological process (BP), molecular function (MF), and cell component (CC). As shown in Figure 3A, 589 GO terms (including 463 BP, 64 CC, and 61 MF) were enriched. The muscle system process was significantly enriched for BP, while for CC, it was was extracellular matrix, and for MF it was the cation transmembrane transporter activity. Furthermore, we analyzed the KEGG pathways of these genes. The results showed 30 KEGG pathways were enriched (Figure 3B), mainly including neuroactive ligand-receptor interaction, Wnt signaling pathway, cell adhesion molecules (CAMs), and renin secretion. The top 20 KEGG pathways are listed in Figure 3B.

### PPI Network Construction and Analysis of Modules

**FIGURE 4:**
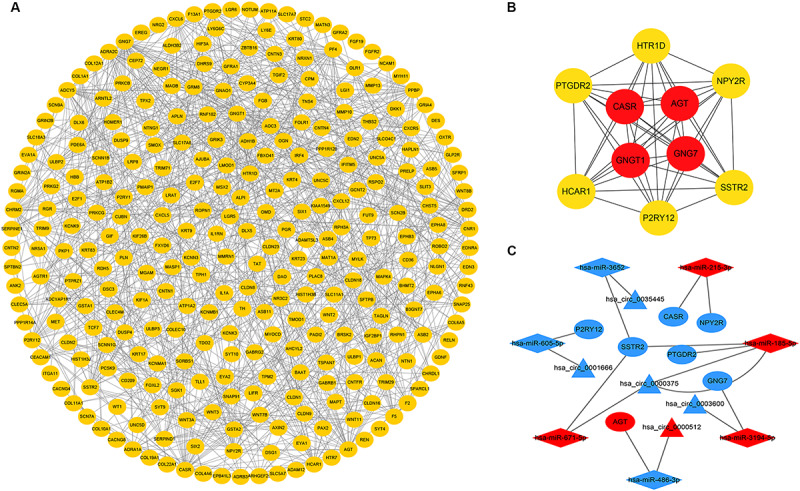
Identification of hub genes from the PPI network and circRNA-miRNA hub gene ceRNA subnetwork. **(A)** a PPI network was constructed from STRING using the 864 common DEmRNAs; **(B)** PPI network of 10 hub genes extracted from A. The node color changes gradually from yellow to red in ascending order according to the log2(foldchange) of genes; **(C)** circRNA-miRNA hub gene ceRNA subnetwork. The nodes highlighted in red indicate upregulation, and the nodes highlighted in blue indicate downregulation. Triangles, diamonds, and ellipses represent circRNAs, miRNAs, and hub genes, respectively.

To further explore the interaction among the 864 common DEGs, we established the PPI network (Figure 4A). The PPI network contained 862 nodes and 828 edges. Ten hub genes were identified based on their MCC score using the Cytoscape software. These screened hub genes were *CASR, AGT, GNGT1, GNG7, HTR1D, PTGDR2, NPY2R, HCAR1, SSTR2, and P2RY12* (Figure 4B). Then, we established a circRNA-miRNA hub gene subnetwork. After excluding the RNAs with the opposite expression of the circRNAs and mRNAs, the subnetwork only contains including five circRNAs (hsa_circ_0035445, hsa_circ_0001666, and hsa_circ_0000375, hsa_circ_0003600, and hsa_circ_0000512), 7 miRNAs (hsa-miR-215-3p, hsa-miR-185-5p, hsa-miR-3194-5p, hsa-miR-671-5p, hsa-miR-486-3p, hsa-miR-3652, and hsa-miR-605-5p), and 7 mRNAs (*P2RY12, SSTR2, CASR, NPY2R, GNG7, AGT, and PTGDR2*) (Figure 4C).

### Establishment of Immune-Related Signature

**FIGURE 5:**
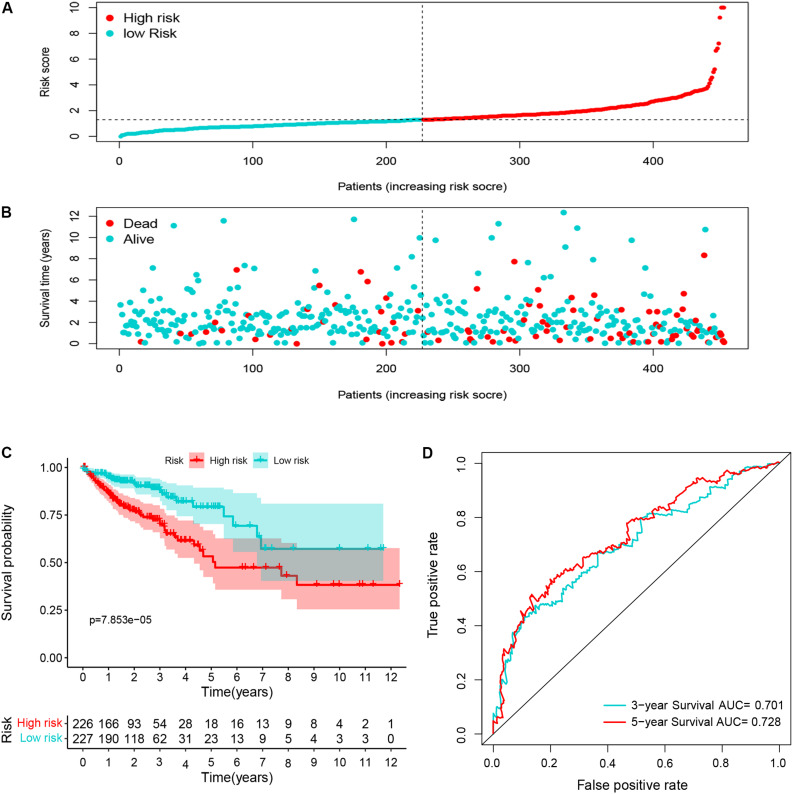
The distribution of risk score **(A)**, survival status **(B)**, Kaplan-Meier curve **(C)**, and ROC curve **(D)** of 7-immune-gene signature.

Ninety-one differentially expressed immune-related genes were initially subjected to univariate analysis. The result showed that 10 immune-related genes were significantly correlated with the OS (*P* < 0.05). Then, the 10 genes were further included in a multivariate analysis. Finally, seven immune-related genes (*SEMA3E, CXCR5, JAG2, STC2, COLEC10, GLP2R*, and *NR3C2*) were identified (Table 2). The different expressions of the 7 genes between CRC and normal colorectal tissues are listed in Figure 5. The risk score = 1.484163 ^∗^ (expression value of *SEMA3E*) + 4.120982 ^∗^ (expression value of *CXCR5*) + 0.033494 ^∗^ (expression value of *JAG2*) + 0.028039 ^∗^ (expression value of *STC2*) – 2.26312 ^∗^ (expression value of *COLEC10*) – 2.55344 ^∗^ (expression value of *GLP2R*) – 0.11606 ^∗^ (expression value of *NR3C2*). The risk score for each patient was calculated and all patients were divided into high (*n* = 226) and low risk group (*n* = 227) based on the median risk score. The Kaplan-Meier log rank test illustrated that there were significant differences in OS (*P* < 0.001) (Figure 5). The AUCs for 3-year and 5-year OS were 0.701 and 0.728, respectively (Figure 5). The risk score, overall survival time, Kaplan-Meier curve, and ROC curve of seven-gene signature were shown in Figure 5.

**TABLE 2:** Seven prognostic mRNAs significantly associated with OS.

**Gene name**	**Coefficient**	**Down/up-regulated**	**Hazard ratio (95% CI)**	***P*-value**
SEMA3E	1.484163	Up	4.41 (2.36−8.24)	<0.001
CXCR5	4.120982	Up	61.62 (10.45−363.49)	<0.001
JAG2	0.033494	Up	1.03 (1.01−1.06)	0.005
STC2	0.028039	Up	1.03 (0.99−1.07)	0.143
COLEC10	−2.26312	Down	0.10 (0−2.51)	0.163
GLP2R	−2.55344	Down	0.08 (0.01−0.54)	0.010
NR3C2	−0.11606	Down	0.89 (0.79−1.00)	0.055

### Construction and Validation of Nomogram

**FIGURE 6:**
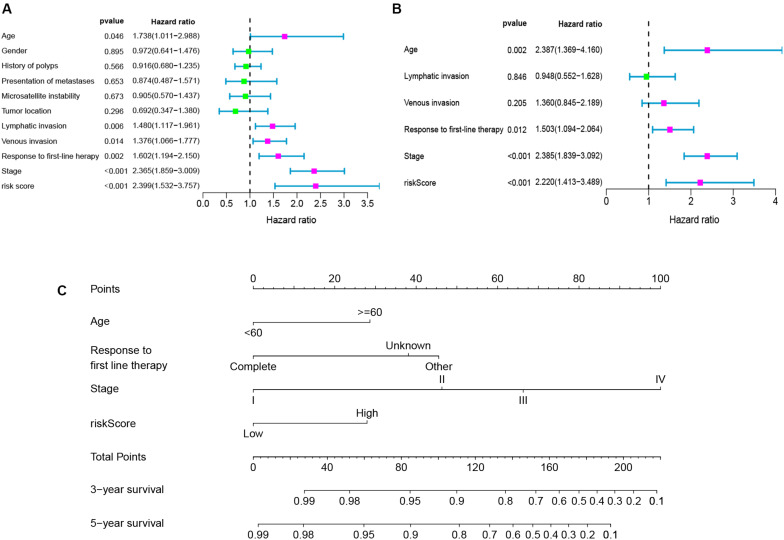
Univariate analyses **(A)**, multivariate analyses **(B)**, and nomogram **(C)** for predicting 3- and 5-year OS of CRC patients.

**FIGURE 7:**
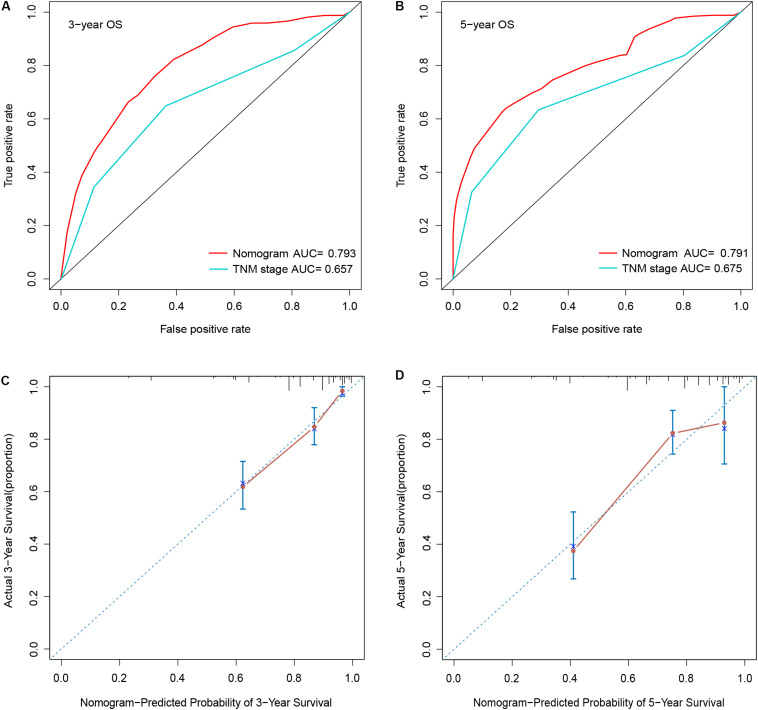
Comparison of the AUCs of the nomogram and AJCC TNM staging system, and calibration curves of the nomogram of patients with CRC. **(A)** 3-year AUC; **(B)** 5-year AUC; **(C)** 3-year calibration curves; **(D)** 5-year calibration curves.

To determine whether the seven-gene signature could be an independent predictor of CRC patients, we performed the univariate and multivariate Cox regression analysis. Age, lymphatic invasion, venous invasion, response to first-line therapy, tumor stage, and risk score were significantly associated with the OS in the univariate analysis (Figure 6A). All the significant factors were selected into the multivariate Cox regression analysis. Only age, stage, response to first-line therapy, and risk score remained statistically significant (*P* < 0.05; Figure 6B). A nomogram was then constructed to predict the 3-year and 5-year OS of CRC patients (Figure 6C). The AUC value for the 3-year (Figure 7A) and 5-year OS (Figure 7B) rates were 0.793 and 0.791, respectively. Moreover, our result demonstrated that the predictive ability of the nomogram was significantly better than the TNM system (Figures 7A,B). The calibration curves for the probability of OS of 3 years or 5 years showed no deviations between the prediction by nomogram and actual reference line (Figures 7C,D).

### Immune Infiltration of Risk Score

**FIGURE 8:**
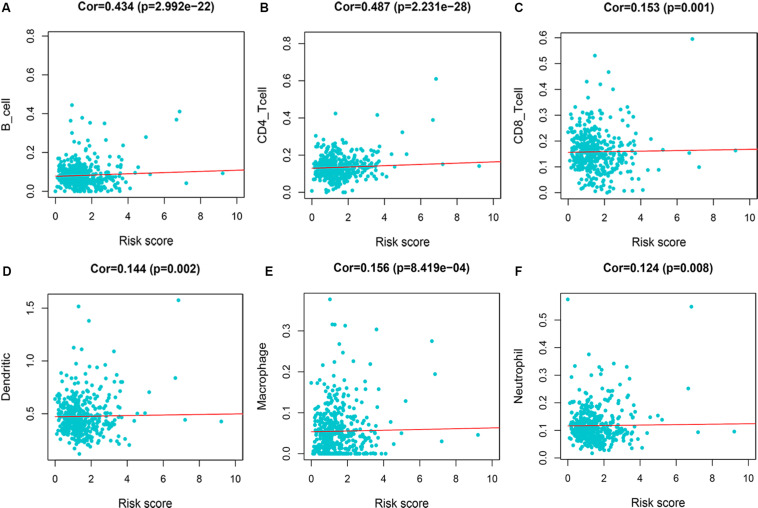
Immune infiltration of 7-immune-gene signature. **(A)** B cells; **(B)** CD4+ T cells; **(C)** CD8+ T cells; **(D)** dendritic cells; **(E)** macrophages; **(F)** neutrophils.

We evaluated the correlation between risk score and immune infiltration level by TIMER online resource. The result indicated that high-risk score was positively correlated with B cells, CD4+ T cells, CD8+ T cells, macrophages, neutrphils, and dendritic cells (*P* < 0.05), prompting a general increase in immune infiltration level (Figure 8).

## Discussion

In this study, we aimed to investigate possible crucial circRNAs impacting on development and progression in CRC patients. To comprehensively analyze the ceRNA network, we collected the RNA expression data from the GEO and TCGA databases, including circRNA, miRNA, and mRNA. We identified 13 DEcircRNAs that may act as a sponge of miRNAs to affect CRC progression via regulating the mRNAs. Based on the joint analysis of multiple databases, we established a CRC-related ceRNA network. Then, we analyzed these 864 DEmRNA functions through the GO database and the KEGG pathway. In addition, PPI networks were also constructed to discover the hub genes. We established circRNA-miRNA hub gene network, including five circRNAs, seven miRNAs, and seven mRNAs. Moreover, we established seven immune-related signatures though immune genes in the ceRNA network. Patients with high-risk had a significantly worse OS than low-risk patients. The Kaplan-Meier curve illustrated that there were significant differences in the OS between high-risk and low-risk groups. The ROC curve demonstrated good performance. Furthermore, we established a prognostic nomogram that combines immune-related risk scores and clinicopathological features. The accuracy of the nomogram for predicting 3-year and 5-year OS was confirmed by the discrimination and calibration plots. Finally, we approved the correlation between risk score and six immune infiltrates.

With the development of high-throughput sequencing technology, more and more circRNAs were found to be abnormally expressed in tumor tissues and to have a critical impact on tumor progression (Wu et al., 2019). Although the functional mechanism of circRNAs remains unclear, accumulating evidence indicates that circRNAs can act as a miRNA sponge to influence the interactions between miRNAs and mRNAs and contribute to the progression of CRC. For example, hsa_circRNA_102958 was found to be upregulated in CRC tissues. Silence of hsa_circRNA_102958 can inhibit CRC growth, migration, and invasion. Further studies revealed that hsa_circRNA_102958 could exert its oncogenic functions by sponging miR-585 and lead to the release of its repression on *CDC25B* (Ruan et al., 2019). Similarly, Yang et al. indicated that circ-ITGA7 was downregulated in CRC tissues. Mechanistically, circ-ITGA7 might sponge miR-3187-3p to increase *ASXL1* level to suppress cell proliferation, migration, and invasion (Li R. et al., 2019). In our study, there were five crucial circRNAs (hsa_circ_0001666, hsa_circ_0035445, and hsa_circ_0000375, hsa_circ_0003600, and hsa_circ_0000512) included in the subnetwork. Afzali and Salimi (2019) constructed a circRNA-miRNA network by microarray analysis and found that hsa_circ_0001666 was down-regulated in breast cancer tissues. However, they did not conduct functional studies. Similarly, hsa_circ_0035445 was highly expressed in gastric cancer tissue by microarray analysis (Shao et al., 2017). However, none of these five circRNAs was reported in the CRC. Therefore, their role in CRC needs to be further verified in future studies.

MiRNAs are types of endogenous noncoding RNA comprising about approximately 19–25 nt long, with widespread participation physiological activity. Studies have found that miRNAs play crucial roles in various pathological processes, and miRNAs have been identified as oncogenes or tumor suppressor genes in regulating cancer progression (Manikandan et al., 2008). MiR-215-3p was found to be downregulated in the 5-FU resistant cell, and high miR-215-3p expression sensitized CRC to 5-fluorouracil-induced apoptosis by regulating the expression of *CXCR1* (Li et al., 2018). Consistent with previous studies, we found a higher expression of miR-215-3p in CRC patients. Zhu et al. (2018) found that lncRNA FOXD2-AS1 could promote proliferation and migration of CRC though interacting with miRNA-185-5p. Della Vittoria Scarpati et al. (2012) confirmed by qRT-PCR that miR-671-5p is significantly upregulated in rectal cancer patients. However, miR-3194-5p, miR-486-3p, and miR-3652 have not been reported in patients with CRC.

In our circRNA-miRNA hub gene subnetwork, seven hub genes with the highest connective degree were selected using the MCC method. The hub genes are *P2RY12*, *SSTR2*, *CASR*, *NPY2R*, *GNG7*, *AGT*, and *PTGDR2*. Some of these genes have been reported to be closely related to CRC, such as *SSTR2*, *CASR*, and *AGT* (Li et al., 2017; Momen-Heravi et al., 2017; Patnaik and Anupriya, 2019). Several studies have found that *SSTR2* is methylated in CRC tissues, suggesting that *SSTR2* may serve as a potential epigenetic marker for early detection of CRC (Li et al., 2017). Momen-Heravi et al. (2017) found that overexpression of *CASR* was correlated with a lower risk of CRC-specific mortality. Martin et al. (2014) indicated that *AGT* epithelial expression was associated with a longer progression free survival, suggesting that *AGT* may be a prognostic marker in metastatic CRC patients treated with chemotherapy and bevacizumab.

KEGG enrichment analyses using DEmRNAs provided an intuitive overview in elucidating the mechanism of CRC. Results suggested that the DEmRNAs were mostly enriched in neuroactive ligand-receptor interaction, the Wnt signaling pathway, cell adhesion molecules (CAMs), and renin secretion. These pathways play vital roles in the development of CRC. For example, Wnt signaling is constitutively overexpressed and is thought to be a crucial step in the development of CRC. Accumulated β-catenin enters the nucleus of colon cancer cells and interacts with T cell factor family transcription factors, leading to activation of downstream target genes of the Wnt pathway and promoting tumorigenesis (Barker and Clevers, 2006). Dong et al. revealed that the secreted V-set and transmembrane domain containing 2A (*VSTM2A*) significantly inhibits the Wnt signaling pathway in colon cancer cells. *VSTM2A* can inhibit the phosphorylation of Wnt signaling co-receptor LDL receptor-associated protein 6 (*LRP6*) and induce *LRP6* endocytosis and lysosomal-mediated degradation, which together lead to the inactivation of Wnt signaling. Maintenance of cell adhesion, promotion, or destruction are particularly important process in colorectal tumorigenesis. Paschos et al. (2009) demonstrated that CAMs play key roles in the progression of CRC and its metastasis to the liver. CAMs are not only related to the detachment of malignant cells from the primary tumor, but also to the attachment of tumor cells to distant tissues. All these findings indicated the development of CRC might be related to these pathways.

Our proposed immune-related signature was significantly associated with OS of CRC patients. The signature constated of seven immune-related genes. Of these seven genes, four are potential risky genes and three are potential protective genes. All four risky genes have been previously reported to have a clear correlation with CRC. Semaphorin 3E (*SEMA3E*) was originally identified as a neurochemical chemotactic agent involved in regulating cell migration, proliferation and angiogenesis (Yong et al., 2016). Studies have indicated that *SEMA3E* plays a role in the regulation of various stages of cancer development. Kang et al. (2016) showed that miR-4282 inhibits CRC cell proliferation by targeting Sema3E by inhibiting its translation or enhancing Sema3E mRNA degradation. Yan et al. (2015) analyzed the expression of *CXCR5* in 236 cases of CRC and adjacent normal tissue, and found that high *CXCR5* expressions could promote the pathogenesis, metastasis and recurrence, suggesting that it can be used as a valuable predictor for metastasis and recurrence of CRC. Similarly, *JAG2* and *STC2* act as oncogenes to promote CRC development and progression through different signaling pathways, such as noncanonical Notch, non-EMT-dependent, and Wnt/β-catenin pathway (He et al., 2019; Li Q. et al., 2019). However, the other three protective genes are rarely reported in CRC. Since a single gene may be predictive of instability in predicting the OS, the signature that integrate the efficacy of seven immune-related genes will show robust predictive power.

In order to establish a relatively accurate prognostic model in CRC patients, a novel prognostic nomogram including immune-related risk score and clinicopathological feature had been proposed in this study, which had displayed favorable predictive capability. In addition, significant correlation between the immune risk score and six immune infiltrating cells were observed. We speculate that there may be a link between the signature and response to immunotherapy. NF-κB is a key component of the immune response and cancer (Karin and Greten, 2005). Studies have shown that inhibition of NF-κBc-Rel attenuates regulatory T cell–mediated immunosuppression and enhances the therapeutic efficacy of anti-PD-1 (Grinberg-Bleyer et al., 2017). Of the seven immune-related genes, three genes are associated with NF-κB activity (Deng et al., 2000; Kermarrec et al., 2019; Zhang et al., 2019). Therefore, it is further shown that the signature was associated with the response to immunotherapy. Nevertheless, the present study included some limitations. First, the circRNA-miRNA-mRNA network was established by bioinformatics analysis. Experimental studies should be conducted to confirm our results. In addition, the performance of the seven-immune-gene signature and nomogram need to be validated by additional data.

## Conclusion

Our study identified five crucial circRNAs and established a circRNA-related ceRNA network involved in CRC, which will assist in understanding the molecular mechanisms underlying the carcinogenesis and progression. Moreover, a seven-immune-based signature were established. The signature might improve the prediction of survival and reflect the immune status and help surgeons to better improve risk stratification and choose the optimal treatments for patients with CRC.

## Data Availability Statement

Publicly available datasets were analyzed in this study. These data can be found in the Cancer Genome Atlas (https://portal.gdc.cancer.gov/) and the NCBI Gene Expression Omnibus (GSE126095).

## Ethics Statement

Ethical review and approval was not required for the study on human participants in accordance with the local legislation and institutional requirements. Written informed consent for participation was not required for this study in accordance with the national legislation and the institutional requirements.

## Author Contributions

WS and TF conceived and designed the original study. WS, JR, and CW collected and analyzed the data. WS, JR, CW, and YG contributed to the interpretation of the data. WS, JR, CW, and YG drafted the manuscript. WS and TF revised the manuscript. All authors saw and approved the final version of the manuscript.

## Conflict of Interest

The authors declare that the research was conducted in the absence of any commercial or financial relationships that could be construed as a potential conflict of interest.

## Footnotes

1 https://www.ncbi.nlm.nih.gov/geo/

2 TCGA, https://cancergenome.nih.gov/

3 https://www.immport.org/shared/genelists

4 http://gb.whu.edu.cn/CSCD/

5 www.cytoscape.org/

6 http://string-db.org/

7 https://cistrome.shinyapps.io/timer/
